# Die Bedeutung der sequenziellen Dermatoskopie zur Vermeidung unnötiger erneuter Exzisionen von rezidivierenden Nävi

**DOI:** 10.1111/ddg.70054x

**Published:** 2026-07-07

**Authors:** Ina Sotiri, Federico Venturi, Elisabetta Magnaterra, Leonardo Veneziano, Aurora Alessandrini, Monika Fida, Emi Dika

**Affiliations:** ^1^ Department of Dermatology and Venereology University Hospital Center “Mother Theresa” Tirana Albania; ^2^ Department of Medical and Surgical Sciences (DIMEC) University of Bologna Bologna Italy; ^3^ Oncologic Dermatology Unit IRCCS Azienda Ospedaliero‐Universitaria of Bologna Bologna Italy

**Keywords:** digitale Überwachung, rezidivierende Nävi, Sequentielle Dermatoskopie, digital monitoring, recurrent nevi, sequential dermoscopy

Sehr geehrte Herausgeber,

Rezidivierende Nävi sind Repigmentierungen, die nach einer unvollständigen Entfernung eines melanozytären Nävus entstehen. Klinisch und dermatoskopisch können sie einem Melanom ähneln. Dies führt häufig zu erneuten Exzisionen und kann bei den Patienten Ängste auslösen. ^1^, ^2^ Wie die Dermatoskopie die Beurteilung pigmentierter Läsionen revolutioniert hat, kann jedoch ein einzelnes Bild möglicherweise nicht die sich im zeitlichen Verlauf entwickelnde Morphologie eines rezidivierenden Nävus erfassen.^3^, ^4^ Nur wenige Berichte haben die Verwendung der sequenziellen Dermatoskopie zur Unterstützung der konservativen Behandlung von rezidivierenden Nävi thematisiert und die Relevanz der sequenziellen Dermatoskopie, allein bei der langfristigen Entscheidungsfindung, wird in der Literatur nach wie vor nur unzureichend behandelt.^5^ Wir stellen zwei anschauliche Fälle vor, in denen die serielle Bildgebung Sicherheit ergab und unnötige chirurgische Eingriffe verhinderte. In beiden Fällen wurden die Patienten von externen Hautkliniken überwiesen. Daher standen keine dermatoskopische Bilder, klinische Fotos oder histologische Präparate zur Überprüfung zur Verfügung. Unsere Beurteilung stützte sich auf die histopathologischen Überweisungsberichte, die in beiden Fällen die Diagnose eines dermalen Nävus bestätigten.

Eine 46‐jährige Frau wurde zur erneuten Exzision einer rezidivierenden pigmentierten Makula im Lendenbereich überwiesen, die acht Monate nach der Entfernung eines dermalen Naevus auftrat. Klinisch zeigte sich eine 15 mm große, unregelmäßige Makula über der Narbe. Die Basisdermatoskopie ergab eine unregelmäßige blaugraue Pigmentierung mit peripheren Streifen, Globuli und hervortretenden Gefäßen (Abbildung 1a). Die dermatoskopische Nachuntersuchung nach drei und sechs Monaten zeigte eine Stabilisierung der Pigmentierung, weniger Globuli und keine Größenveränderung (Abbildung 1b). Auf der Grundlage dieser sequenziellen Überwachung deutete der Gesamteindruck auf einen rezidivierenden Nävus hin, und eine fortgesetzte Überwachung wurde empfohlen.

**ABBILDUNG 1:**
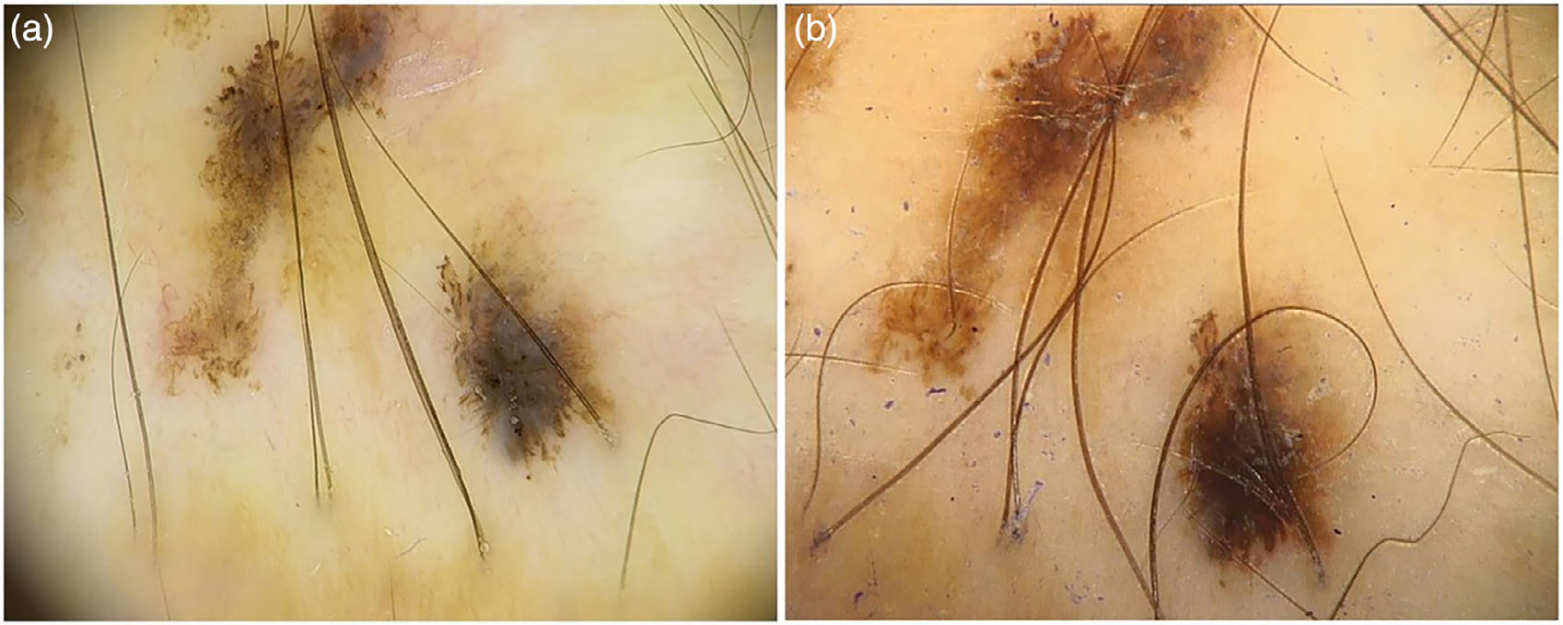
Sequenzielle dermatoskopische Bilder des rezidivierenden Nävus einer 46‐jährigen Frau mit einer rezidivierenden pigmentierten Läsion im Lendenbereich nach Exzision eines dermalen Nävus. (a) Basisdermatoskopie, mit unregelmäßiger blaugrauer Pigmentierung, peripheren Streifen und Globuli. (b) Nachuntersuchung nach 3 Monaten, die eine Stabilisierung der Pigmentierung, eine Verringerung der peripheren Globuli und eine unveränderte Größe der Läsion zeigt, was mit einem gutartigen rezidivierenden Nävus übereinstimmt.

Eine 48‐jährige Frau stellte sich mit zwei repigmentierten Läsionen an der rechten Schulter vor, die sich sechs Monate nach der histologisch bestätigten Entfernung von dermalen Nävi durch Shave‐Biopsien entwickelt hatten. Die erste Läsion, eine 5 mm große braune Makula, zeigte ein braunes Pigmentnetzwerk mit peripheren Punkten und Globuli (Abbildung 2a). Nach drei Monaten war die Läsion unverändert, nach sechs Monaten war sie kleiner und homogener geworden, mit einem weniger ausgeprägten Pigmentnetzwerk (Abbildung 2b, c). Die zweite Läsion, eine 3,6 mm große Papel, wies ein braunes Netzwerk mit zentrifugalem Wachstum und radialen Streifen auf, die auf die Narbe beschränkt waren (Abbildung 2d). Auch dieses Erscheinungsbild war auf den ersten Blick besorgniserregend. Nach drei Monaten war die Pigmentierung weniger ausgeprägt, und nach sechs Monaten hatte sich die Läsion verkleinert und die Streifen waren vollständig verschwunden (Abbildung 2e, f). Die sequenzielle Überwachung bestätigte das gutartige Verhalten und vermied somit zwei unnötige erneute Exzisionen.

**ABBILDUNG 2:**
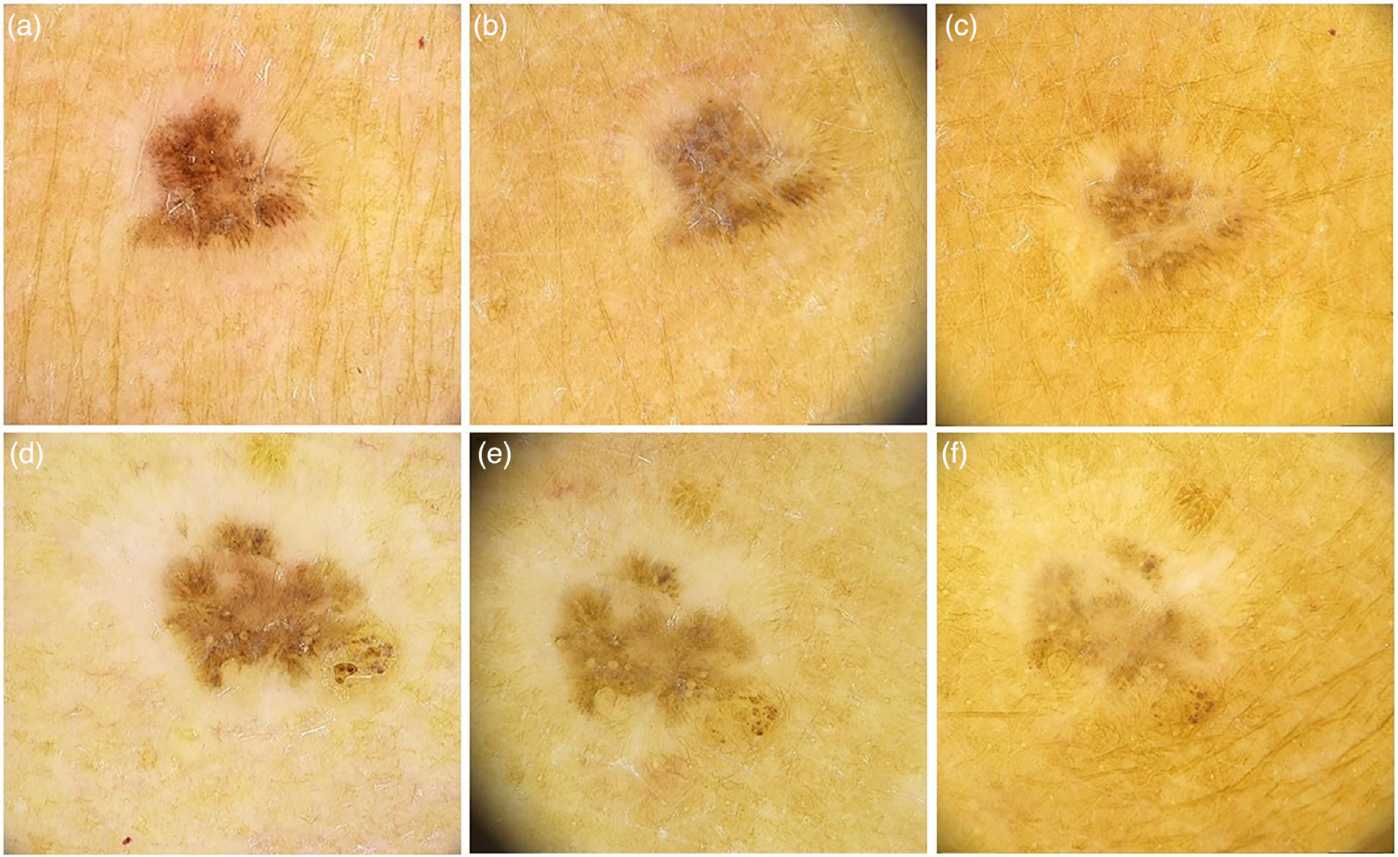
Sequenzielle dermatoskopische Entwicklung von zwei rezidivierenden Nävi bei einer 48‐jährigen Frau mit zwei repigmentierten Läsionen an der rechten Schulter nach Entfernung der dermalen Nävi. Ausgangsbild einer 5 mm großen braunen Makula mit einem braunen Pigmentnetzwerk, mit peripheren Punkten und Globuli (a), verminderter Netzwerkpräsenz nach 3 Monaten (b) und Verringerung der Pigmentierung mit einem homogeneren Muster nach 6 Monaten (c). Ausgangsbild einer 3,6 mm großen Papel mit einem braunen Netzwerk und radialen Streifen, die auf die Narbe beschränkt sind (d), teilweises Verblassen der Pigmentierung nach 3 Monaten (e) und vollständige Rückbildung der Streifen und homogene Narbenpigmentierung nach 6 Monaten (f).

Nach sechs Monaten zeigten alle Läsionen ein stabiles oder regressives Verhalten, und die Patienten wurden wieder in die routinemäßige jährliche Nachsorge integriert.

Die vorgestellten Fälle verdeutlichen die diagnostische Herausforderung bei rezidivierenden Nävi und unterstreichen den klinischen Nutzen der sequenziellen Dermatoskopie bei der Unterscheidung zwischen einer gutartigen Repigmentierung und einem malignen Rezidiv. Zu einem bestimmten Zeitpunkt wiesen beide Läsionen Merkmale auf, die häufig mit Melanomen assoziiert werden, wie Asymmetrie, unregelmäßige Pigmentierung und Streifen.^2^ Die Analyse dieser Muster im Verlauf der Zeit ergab jedoch eine Stabilisierung oder sogar eine Auflösung der atypischen Merkmale, was das gutartige Verhalten der hier vorgestellten rezidivierenden Nävi bestätigte. Unsere Ergebnisse stimmen mit früheren Beobachtungen überein, die darauf hindeuten, dass viele atypische dermatoskopische Merkmale bei rezidivierenden Nävi eher auf entzündliche oder reparative Veränderungen im Narbengewebe als auf eine maligne Transformation zurückzuführen sind.^6^, ^7^, ^8^ Die meisten bisherigen Veröffentlichungen haben sich auf die Histopathologie und die einmalige Dermatoskopie bei rezidivierenden Nävi konzentriert. Dies ist zwar bei der Unterscheidung zwischen reaktiven Hyperpigmentierungen und rezidivierenden melanozytären Neoplasien hilfreich, kann aber nur begrenzt als Leitfaden für die longitudinale Beurteilung dienen. ^2^, ^7^ Die sequenzielle Dermatoskopie führt eine dynamische Perspektive ein und bietet den Ärzten eine zeitliche Dimension zur Beurteilung des zukünftigen Verhaltens von Läsionen. Dieser Ansatz erleichtert die Unterscheidung zwischen einem aktiven Nachwachsen und narbenbedingtem Umbau und trägt dazu bei, Überbehandlungen, chirurgische Morbidität und Ängste der Patienten zu reduzieren.^9^, ^10^, ^11^ Zusammenfassend lässt sich sagen, dass rezidivierende Nävi dermatoskopische Merkmale aufweisen können, die einem Melanom ähneln und oft durch zugrunde liegende Entzündungs‐ und Wundheilungsprozesse beeinflusst werden. Die sequenzielle Dermatoskopie ist ein kostengünstige, nicht‐invasive Methode zur Überwachung der Läsionsentwicklung und ermöglicht eine sichere konservative Behandlung. In einem breiteren klinischen Kontext können optimierte Strategien zur Beurteilung von Nävi, wie z. B. die Schätzung der Gesamtzahl der Nävi am Körper durch Zählen der Nävi am rechten Arm, ebenfalls zur Risikostratifizierung und Entscheidungsfindung in der Primärversorgung beitragen.^12^ Die Einbeziehung einer bildgebenden Diagnostik in die klinische Praxis, die auf dem zeitlichen Verlauf basiert, kann unnötige Exzisionen reduzieren und sowohl die Diagnosegenauigkeit als auch die Zufriedenheit der Patienten steigern.

## INTERESSENKONFLIKT

Keiner.
